# New Insights Into the Pathologic Roles of the Platelet-Activating Factor System

**DOI:** 10.3389/fendo.2021.624132

**Published:** 2021-03-15

**Authors:** Jeffrey B. Travers, Joyce G. Rohan, Ravi P. Sahu

**Affiliations:** ^1^ Department of Pharmacology & Toxicology, Boonshoft School of Medicine at Wright State University, Dayton, OH, United States; ^2^ Department of Dermatology, Boonshoft School of Medicine at Wright State University, Dayton, OH, United States; ^3^ Dayton Veterans Administration Medical Center, Dayton, OH, United States; ^4^ Naval Medical Research Unit Dayton, Environmental Health Effects Directorate, Wright Patterson Air Force Base, OH, United States

**Keywords:** platelet-activating factor (PAF), oxidized glycerophosphocholine, skin, central nervous system, cancer, inflammation, ultraviolet - B, microvesicle particles

## Abstract

Described almost 50 years ago, the glycerophosphocholine lipid mediator Platelet-activating factor (PAF) has been implicated in many pathologic processes. Indeed, elevated levels of PAF can be measured in response to almost every type of pathology involving inflammation and cell damage/death. In this review, we provide evidence for PAF involvement in pathologic processes, with focus on cancer, the nervous system, and in photobiology. Importantly, recent insights into how PAF can generate and travel *via* bioactive extracellular vesicles such as microvesicle particles (MVP) are presented. What appears to be emerging from diverse pathologies in different organ systems is a common theme where pro-oxidative stressors generate oxidized glycerophosphocholines with PAF agonistic effects, which then trigger more enzymatic PAF synthesis *via* the PAF receptor. A downstream consequence of PAF receptor activation is the generation and release of MVP which provide a mechanism to transmit PAF as well as other bioactive agents. The knowledge gaps which when addressed could result in novel therapeutic strategies are also discussed. Taken together, an enhanced understanding of the PAF family of lipid mediators is essential in our improved comprehension of the relationship amongst the diverse cutaneous, cancerous, neurologic and systemic pathologic processes.

## Introduction

The term “platelet-activating factor” was first given by Benveniste and colleagues in their landmark Journal of Experimental Medicine manuscript in 1972, to a biochemical activity released by activated basophils which caused platelets to aggregate (1). This activity (PAF) was subsequently determined to be a class of glycerophosphocholines (GPC) with 1-hexadecyl-2-acetyl-GPC being amongst the most potent (2, 3). Though PAF has been demonstrated to have multiple biological activities due to a binding at high picomolar-low nanomolar concentrations to single G-protein coupled receptor widely expressed (4, 5), the term Platelet-activating factor has remained. Indeed, the PAF family of lipid mediators have been implicated in pro-inflammatory processes ranging from asthma to sepsis to ultraviolet radiation (UVR) responses. Administration of PAF results in an acute inflammatory response, yet also can generate immunosuppressive effects *via* upregulation of regulatory T cells (6–9). A recent PubMed search indicates that more than 14,000 publications involve PAF, which attests to the large body of information available on this lipid mediator.

The synthetic pathways for PAF have been extensively reviewed (5, 10). The major pathway associated with cellular stimuli is the remodeling pathway. Cellular activation resulting in increased intracellular calcium levels induces phospholipase A_2_ (often group IVA cytosolic cPLA_2_) activation which releases an unsaturated fatty acid from the *sn-2* position of a GPC, with the released fatty acid often a substrate to form eicosanoids. The lyso GPC species then is acetylated using acetylCoA by an acetyltransferase (10) to form PAF. Of interest, the PAF receptor (PAFR) is a potent stimulus for enzymatic PAF synthesis *via* this pathway, indicating a feed-forward system (11). The limit on enzymatic PAF synthesis appears to be the substrate as well as amounts of acetylCoA available. Once produced, PAF is quickly broken down by cell- and serum-associated PAF acetylhydrolases (PAF-AH) (12). Thus, PAF is a highly potent mediator whose synthesis and degradation are tightly regulated.

In contrast to the highly regulated enzymatic pathways, PAFR agonists can also be formed in response to reactive oxygen species (ROS) *via* the direct attack of free radicals on the *sn-2* long-chained unsaturated fatty acids in the GPC (13–15). Oxidation of the esterified fatty acyl residue can introduce oxy functions, bond rearrangements and can result in the fragmentation of carbon-carbon bonds *via* β-scission. This process can result in a large number of phospholipid reaction products, to include some that exert PAFR agonistic activity. Unlike the tightly regulated enzymatic processes, this non-enzymatic process producing oxidized GPC (ox-GPC) with PAFR activity is dependent upon amounts of GPC substrate, pro-oxidants and antioxidant defenses. It should be noted that the majority of ox-GPC species have not been structurally characterized to allow quantitation using mass spectrometric techniques. Hence, we believe that the most accurate manner to measure PAF is *via* its biochemical effects such as intracellular calcium mobilization responses or release of cytokines such as IL-8 in genetically engineered cell lines with/without the PAFR (14, 16–18).

Once PAF is generated, it can either reside in the cellular membranes and potentially act upon the cell itself or to neighbor cells in juxtacrine fashion (19, 20). Some cell types, in particular monocytes, neutrophils and keratinocytes have been demonstrated to release PAF to allow it to exert effects away from the host cell. The exact mechanisms by which cells release PAF is as yet unknown, however, our group has recently demonstrated that a keratinocyte cell line can generate subcellular microvesicle particles (MVP; see ref (21–23) for recent reviews) released from budding from the plasma membrane which contain PAFR agonistic activity (24, 25). As PAFR activation results in both MVP release as well as PAF generation, this results in the potential linkage of the two processes. Presumably, traveling in an MVP might afford protection from degradation by PAF-AH in comparison to being free or protein-bound in tissue fluids. As MVPs merge with target cells, this could place PAF back into a target cell membrane. It should be noted that recent evidence from x-ray crystallography of the PAFR indicates that helix VIII appears to cover the ligand-binding site (26). This novel structural finding could be suggestive that optimal PAFR agonists bind to the receptor while residing in the plasma membrane, rather than accessing the binding site extracellularly. This finding might also provide an explanation for the low affinity of all known PAFR antagonists in comparison to native PAF ligand (27). Of importance, MVP release from many stimuli (including PAF) are dependent upon the lipid enzyme acid sphingomyelinase (aSMase) (25, 28). As aSMase inhibitors including imipramine and other molecules in the tricyclic anti-depressive class of molecules are available (29), this could potentially result in pharmacologic modulation of PAF release. This adds a potential adjunctive pharmaceutical strategy in addition to the use of PAFR antagonists.

## PAF and Disease Processes

Because elevated PAF levels can be measured in many diseases, and exogenous PAF can mimic many aspects of disorders, PAF has been implicated in many processes. However, no actual diseases have been demonstrated to be due entirely to the presence or lack of the PAF system. The picture that is emerging is that the PAF system appears to serve as a modulator of pathologic processes. There are three areas that we would like to focus this review upon- the role of PAF in cancers and cancer therapy responses, central nervous system pathologies, and the effects of ultraviolet B radiation. Given that the skin is a complex organ that has epithelial, mesenchymal, immunologic, and neuronal components, all of these areas link to the overall theme of the epilipidome. Moreover, the three areas are connected to what we believe is a common three-part process. First, ROS from various agents generate small levels of PAFR agonists. Second, these PAFR agonists act upon the PAFR resulting in cellular activation and generation of additional PAF enzymatically, potentially allowing a PAFR amplification response. Finally, aSMase activation results in the formation and release of MVP carrying PAFR agonists to other sites. Activation of PAFR in target organs then mediate further pathology. This process provides several therapeutic targets to include antioxidants, PAFR antagonists, aSMase inhibitors as well as agents that block down-stream effects such as cyclooxygenase-2 (COX-2) inhibitors.

## PAF and Cancer and Cancer Therapies

### Evidence Linking PAF System to Cancers

The ability of the PAF-PAFR signaling to induce a robust systemic pro-inflammatory, pro-proliferative, and delayed immune suppressive responses, implicated in various pathological conditions, rationalized its exploration in cancer development as many malignant cells were identified to express PAFR (3–5, 30). Notably, studies by Im and colleagues provided the first report demonstrating that a single systemic administration of PAF can augment IL-1α and TNF-α-induced increased pulmonary metastasis of murine B16F10 melanoma cells in syngeneic C57BL/6 hosts, in a process blocked by the PAFR antagonist BN50739 (31). The critical role of the PAF-PAFR signaling in melanoma tumorigenesis was also supported by the phenotype of the PAFR-overexpressing transgenic mice that exhibited keratinocyte hyperplasia, which was accompanied by hyperpigmentation and increased number of dermal melanocytes in the ear and tail with subsequent development of melanocytic tumors (32, 33). Since the PAFR is expressed in keratinocytes but not melanocytes (34), it is presumed that the melanocytic tumors are in response to inappropriate expression of the PAFR which resulted in a proliferative response. Biancone and colleagues also evaluated the role of the PAF-metabolizing enzyme PAF-AH in melanoma tumor development (35). C57BL/6 mice implanted with PAF-AH-overexpressing B16F10 melanoma cells exhibited significantly decreased tumor vascularization and growth, as well as increased survival compared to the mice harboring PAF-AH-deficient B16F10 tumors (35). These studies provided the rationale to further define the contributions of tumoral versus host PAFR signaling in melanoma development.

Given that ectopic PAF-AH expression in KS-Imm human Kaposi’s sarcoma cells, or B16F10 melanoma cells resulted in reduced neoangiogenesis and tumor growth in their respective SCID and C56Bl/6J hosts, and that IFNγ-stimulated PAF synthesis enhanced the invasiveness of F10-M3, a clone of B16F10 melanoma line (35, 36), the direct evidence of tumoral PAFR in melanoma growth remained unclear as unlike many human melanoma cell lines (37–40), murine B16F10 cells do not express PAFR (41, 42). To address this question, our group generated PAFR-expressing B16F10 melanoma cells and demonstrated that regardless of the tumoral-PAFR expression, systemic administration of a PAFR agonist, CPAF resulted in increased growth of melanoma tumors in wild type (WT) mice, but not in PAFR-deficient (PAFR-KO) counterparts (43). Using environmental UVB exposure that generates PAF-agonists (to mirror systemic immunosuppressive model), we observed that similar to systemic CPAF injection, cutaneous UVB radiation also significantly increased the growth of PAFR-deficient, parental B16F10 tumor xenografts in WT hosts, and can be blocked by antioxidants supplementation (43). Importantly, this UVB-mediated increased growth of melanoma tumors was not seen in PAFR-KO hosts (43). Along similar lines, we have also shown that host activation of the host PAFR signaling augments the in-vivo growth and metastatic ability of murine Lewis lung carcinoma cells, yet these effects were not seen in PAFR-KO hosts (44). These studies support an important role of the host PAFR signaling in favoring the development and progression of melanoma and lung tumors.

### PAFR Expression in Tumor Proliferation and Clinical Significance

As most malignant cells of murine and human origins such as the Kaposi’s sarcoma, breast, prostate, lung, esophageal squamous cell carcinoma, ovarian, and pancreatic cancers express PAFR (40, 45–52), several studies have evaluated its relevance using various experimental *in vitro* models, as well as in clinical settings of cancer patients. Data from *in vitro* cellular models indicate that regardless of the anatomical origins, genetic backgrounds or the mechanisms involved, activation of tumoral-PAFR or PAFR overexpression accelerate the proliferation, aggressiveness, migration and invasion compared to respective control cells in various cancer models (45, 49, 50). Of note, multiple tumor cell lines expressing PAFR have been shown to produce more PAF or undergo increased PAFR expression in response to various stimuli including multiple growth factors and therapeutic agents (39, 40, 42). These lines of evidence also suggest that tumoral-PAFR expression could directly modulate the *in vivo* tumor growth *via* inducing systemic immunosuppressive effects mediated by more enzymatic PAF production by positive feed-forward mechanisms. Of significance, high tumoral-PAFR expression has also been detected in clinical settings of primary as well as lymph node metastatic tumors compared to the matched normal tissue (49, 50). High levels of tumoral PAFR expression was found to be positively correlated with increasing tumor stages, tumor status, tumor invasiveness, and poor prognosis in lung and esophageal squamous cell carcinoma patients (49, 50). Importantly, patients with high PAFR-expressing tumors experienced significantly decreased overall survival compared to the patients with low tumor PAFR expression (49, 50). Moreover, increased PAF concentrations were also detected in tumor samples of esophageal squamous cell carcinoma patients compared to matched adjacent normal tissue (50). These studies suggested the translational significance of tumoral PAFR expression in impacting not only tumor progression but also affecting the prognosis and overall survival of cancer patients.

### PAFR Activation Blocking Anti-Tumor Immune Responses

Immune and non-immune factors including inflammatory milieu within the tumor microenvironment play significant roles in fostering tumor growth, angiogenesis, and metastatic progression (53). Given that PAFR activation is critical in both acute inflammatory and delayed systemic immunosuppressive effects, studies including ours have assessed its function in anti-tumor immune responses. Among various immune cell types, macrophages have been recognized for their contributions not only in phagocytosis but also in pathological conditions such as cancer. Macrophages express various receptors including for immunoglobulin (e.g., IgG), endotoxin, phosphatidylserine (PS) etc., which get stimulated upon the engulfment of microbial organisms or their products to then mediate proinflammatory signals. However, when apoptotic bodies are presented, PS interaction with PS-receptor (PSR) on macrophages induce anti-inflammatory signal (54, 55). Importantly, the published reports have also shown that the clearance of apoptotic cells by macrophages induces their differentiation into a regulatory phenotype possessing immune suppressive function. The scavenger receptor CD36 expressed on macrophages, binds to oxidized low density lipoproteins (oxLDL) consisting of phospholipids, which also act as ligands for apoptotic cells. Thus, CD36 mediates apoptotic cell recognition by macrophages and facilitates its clearance (54, 55). As oxLDL mediated effects were found to be blocked by PAFR antagonists, studies by Oliveira et al., have demonstrated that blockade of PAFR or CD36 inhibited apoptotic cell phagocytosis (i.e., efferocytosis) by bone-marrow derived murine macrophages (56). These studies have also shown that this efferocytosis increased the colocalization of CD36 and PAFR in the plasma membrane of macrophages (56). Overall, the data indicate that apoptotic cell phagocytosis requires the engagement of both CD36 and PAFR in lipid raft, which induces macrophage differentiation into a regulatory phenotype.

Earlier studies have evaluated the role of PAF in macrophage regulation *via* measuring its spreading ability *ex vivo*. Macrophages were isolated from the peritoneal cavity of mice bearing Ehrlich ascites tumor (EAT) tumors and treated with or without PAF antagonist and added over glass coverslips. Increased spreading of macrophages was observed within a shorter period after tumor cell implantation. However, as tumors continued to grow, the spreading of macrophages derived from the normal (vehicle-treated) mice decreased, but macrophages from PAF antagonist-treated mice maintained the elevated levels of spreading (57).

Systemic treatment of PAF antagonists not only reduced the *in vivo* growth of EAT but also restored the spreading capability of macrophages (57). Importantly, PAFR antagonists have also been shown to decrease the growth of B16F10 tumors as well as the number of tumor-associated regulatory immunophenotypes expressing galectin-3 (58). Based upon the stimuli and tumor microenvironment, macrophages can acquire pro-inflammatory (M1) or anti-inflammatory (M2) phenotypes. These findings were supported by a recent report demonstrating that the *in vivo* tumor growth of PAFR-expressing TC-1 carcinoma or PAFR-deficient B16F10 melanoma were significantly reduced in PAFR-KO compared to their WT counterparts. Interestingly, the reduced growth of these tumor types in PAFR KO mice was accompanied by increased infiltration of Gr-1+ neutrophils and CD8+ T cells in B16F10 tumors, and CD4+ T cells in TC-1 tumors (59). In addition, both tumor types from PAFR KO mice exhibited high frequency of CD11c+ M1 and decreased frequency of CD206+ M2 macrophages consistent with higher iNOS, lower arginase activity and IL-10 expression levels as compared to the tumors implanted into WT mice (59). Overall, these findings suggested that endogenous PAF-like molecules bind with macrophages expressing PAFR, which then acquire more M2-like phenotype (TAMs) in the tumor microenvironment to favor tumor promotion. Importantly, a novel macrophage phenotype (i.e., Mox) has also been identified from oxidized phospholipid-treated murine macrophages, which possess distinct genetic profiles characterized by overexpression of Nrf2-mediated redox-regulatory genes, decreased phagocytic and chemotactic capabilities (60). These studies indicated that Mox macrophages could play critical roles in the development of development of atherosclerotic lesion as well as chronic inflammation.

Notably, dendritic cells have been shown to express PAFR, and its stimulation was found to result in increased production of cytokines such as IL-10 and the prostaglandin PGE_2_ associated with the regulatory phenotype in a process blocked by PAFR antagonists (61). Our studies have also supported the involvement of immune cells and PAFR in regulating the tumor growth by demonstrating that PAFR activation did not appreciably augment the growth of B16F10 melanoma tumors in immunocompromised SCID mice (43). As other prominent suppressive immunophenotypes which play critical roles in host anti-tumor immune response are regulatory T cells (Tregs), we have shown that increased growth of B16F10 tumors mediated *via* UVB-PAF agonists in syngeneic hosts was correlated with upregulation of tumoral Tregs compared to the sham-treated mice (43). Importantly, depletion of Tregs *via* anti-CD25 Abs or neutralizing Abs against IL-10, a cytokine secreted by immunosuppressive phenotypes including Tregs significantly blocked both UVB- and CPAF-mediated increased growth of B16F10 tumors compared to control groups of mice injected with isotype control Abs (43). Similarly, these effects are blocked by COX-2 inhibitors which appear critical for PAF-mediated Treg generation (18, 42). Myeloid-derived suppressor cells (MDSCs), a heterogeneous group of immature myeloid cells have also been shown to favor tumor development *via* mechanisms including the recruitment of other suppressive immunophenotypes into the tumor microenvironment (62). Given that murine MDSCs express CD11b and Gr-1 surface markers, and that their depletion have been explored as potential therapeutic approaches against solid tumors (reviewed in ref. 62), we evaluated its role in mediating PAFR-dependent tumor growth. Our studies found that UVB- or CPAF-mediated increased growth of B16F10 melanoma tumors was blocked by depleting MDSCs (via systemic injection of anti-Gr-1 Abs) (63). Overall, these studies indicated the relative contributions of several immune cell types in favoring tumor growth.

### Multiple Therapies That Kill Tumor Cells Generate PAF

That dying tumor cells could generate PAF ligands provided the premise to explore the significance of PAFR signaling in the therapeutic efficacies of anti-cancer agents with known cytotoxic effects. Studies including ours have shown that multiple tumor lines including melanoma, lung, lymphoma, pancreatic, and nasopharyngeal carcinomas generate oxidized PAF agonists in response to chemotherapeutic agents or radiation therapy, with increased levels were detected in PAFR-expressing tumor cells (41, 42, 52, 64). Importantly, increased tumoral PAFR expression has also been detected upon the treatment of chemotherapy and radiation therapy (39, 64). These findings led to the hypothesis that systemic generation of PAF agonists *via* these therapies could tolerize the tumor bearing mice due to their ability to induce systemic immunosuppression, and thus, can impact their therapeutic efficacies. Notably, one of the major challenges in the medical oncology field is to define the mechanisms involved in inducing tumor resistance to the ongoing therapeutic options for cancer treatment, with the focus of devising novel approaches to improve their effectiveness. To address this clinically relevant question, ours and Sonia Jancar’s group have evaluated the potential significance of this “bystander effect” generated by chemotherapeutic agents and radiation therapy using murine cutaneous melanoma or squamous cell carcinoma (SCC) tumor lines (41, 42, 64).

Consistent with the systemic immunosuppressive model, we implanted two tumors into the flanks of mice, where one tumor is treated with chemotherapy or radiation therapy and their responses were evaluated on the growth of secondary-untreated tumors. Our studies demonstrated that treatment of one tumor resulted in increased growth of secondary tumor in a PAFR-dependent manner in a process blocked by systemic administration of antioxidants, cyclooxygenase type 2 (COX-2) inhibitors or depleting antibodies against Tregs (41, 42). Consistent with our findings, Jancar’s group have shown that co-injection of irradiated TC-1 cells with TC-1 expressing luciferase (TC-1 fluc+) in syngeneic hosts, or human PAFR-expressing KBP cells with irradiated PAFR-deficient KBM cells in immunocompromised mice resulted in increased tumor growth compared to the mice co-injected with unirradiated TC-1 cells with TC-1 fluc+ or KBM cells with irradiated KBM cells (64). In another report, the same group investigated the involvement of PAFR in tumor cell survival following radiation therapy (65). They observed a dose-dependent increased expression of the PAFR, increased generation of PAF agonists, and secretion of PGE_2_ after radiation therapy in keratinocytes (HaCaT), cervical cancer (C33, SiHa, HeLa), and squamous carcinoma (SSC78 and SSC90) cell lines compared to their respective unirradiated controls (65). Treatment with PAFR antagonist CV3988 pre-radiation therapy reduced PGE2 secretion and increased tumor cell death compared to untreated controls, indicating the tumor cells generate PAF agonists to protect themselves from cell death (65).

Importantly, our studies have also detected an increased level of PAFR activity in the perfusates collected post-chemotherapy compared to pre-chemotherapy in melanoma patients using the isolated limb chemoperfusion (42). Higher concentrations of tumoral PAF were measured in post-radiation compared to pre-radiation therapy treated basal cell carcinoma (BCC), bladder cancer, or pseudo lymphoma patients (41). Increased tumoral PAFR expression was detected in post-radiation therapy treated compared to untreated cervical invasive carcinoma patients (64). These studies suggest that PAF agonists generated *via* these therapeutic agents impede treatment efficacies in a PAFR dependent manner, and that PAFR serves to mediate pro-survival responses to these agents.

### Potential Pharmacologic Strategies

Several groups including ours have proposed PAFR as a promising target to not only inhibit tumor growth but also to increase the efficacy of therapeutic agents. This hypothesis has been tested in multiple experimental models demonstrating that genetic blockade of the PAFR (via studies in PAFR KO mice or PAFR shRNAs) or pharmacologic PAFR antagonists significantly reduced tumor burden, increased murine host survival, as well as augmented the effectiveness of therapeutic agents compared to their respective control groups (39, 41–44, 49, 50, 58, 64, 65). While multiple structurally different but specific PAFR antagonists have been shown to exert promising effects against various experimental tumor models (39, 58, 64, 65), none of these agents have been explored in clinical settings of cancer patients. Importantly, the structural analysis as well as anti-inflammatory effects of several organic compounds (natural and synthetic) and different classes of metal-based inhibitors of PAF have been tested with the focus if these agents could exert anti-cancer properties (66). The authors observed that while these metal-based inorganic compounds possess a very promising class of anti-PAF and anti-inflammatory drugs, the rhodium(III) PAF inhibitor Rh-1 exhibited the moderate cytotoxicity in HEK 293 cell lines, which corroborates with its increased anti-inflammatory action (66). Overall, these studies provided the rationale of designing and exploring a new class of metal-based inhibitors of PAF. Another recent review has summarized the effects and outcomes of major synthetic PAF antagonists tested in clinical trials against several disease pathologies (67). The overall outcomes of these clinical studies are that majority of the synthetic PAF antagonists exerted no significant effects in reducing the clinical symptoms in patients, indicating the exploration of other compounds for their effects as potent inhibitors of PAF or PAFR (67). Thus, other strategies such as the inhibition of ox-GPC formation using antioxidants or the PAFR-mediated immunomodulatory effects using COX-2 inhibitors could be alternative strategies. Finally, the role of MVPs released from tumor cells as the potential source of PAFR agonists could provide an alternative pathway which could be amenable to aSMase inhibitors.

## PAF in CNS Function and Pathology

### Evidence for PAF System in CNS Pathologies

At present, it is unclear whether the PAF system plays an important functional role in the nervous system. Though PAF injection into the skin has been reported to be painful (68–71), much of the evidence linking the PAF system and neuropathology is derived from studies of the central nervous system (CNS). Previous work reported that PAFR agonists can increase intracellular calcium concentration (72), inhibit acetylcholine release in hippocampal slices (73), enhance catecholamine release from cultured chromaffin cells (74), inhibit ionotrophic GABA receptor activity in hippocampal neurons (75), and enhance glutamate release from primary hippocampal cultures (76). There is conflicting evidence with regard to PAF’s role in long term potentiation (LTP), an example of synaptic plasticity in which a synapse enhances its strength typically resulting from high frequency stimulation. One study reported PAF treatment can induce LTP in hippocampal slices at similar potentiation level as that induced by high frequency stimulation (77). However, these data could not be replicated in a later study, in which they demonstrated that PAF alone could not induce LTP in hippocampal slices (78).

Although the role of PAF in CNS function is unknown, the PAF system has been implicated in multiple CNS pathological states. Elevated PAF levels have been detected in and appear to correlate with severity of several CNS diseases. The association of elevated PAF with Alzheimer’s Disease (AD), multiple sclerosis (MS), cerebral infarction, cerebral ischemia-reperfusion injury, and spinal cord injury has been recently reviewed (79). Moderate increases of 20% in PAF were measured in healthy subjects as they age over a 7.5 year period, whereas a greater than 60% average increase in blood PAF levels was detected in AD patients (80). Similarly, patients suffering from MS have elevated CSF as well as plasma PAF levels as compared to healthy subjects (81). Increase in hippocampal PAF levels was observed after evoked seizures in a mouse model of temporal lobe epilepsy (82). Ischemia and traumatic brain injury (TBI) have also been shown to result in significant production of PAF contributing to the pathophysiological events following ischemia or TBI (83–86).

In addition to increases in PAF levels, a reduction in PAF-AH activity was observed in PD (87) and MS patients (88). PAF-AH is an enzyme that degrades PAF and has been shown to be upregulated in stroke patients likely as a response to elevated blood PAF and PAFR agonist levels (86, 89). There is no clear explanation for the reduction in PAF-AH measured in PD and MS patients that were previously reported. The authors suggest that there may additional lipid oxidation products during PD and MS progression that may act to inhibit the PAF-AH and thus inducing and/or augmenting the elevated PAF levels (87).

### PAFR Activation and CNS Toxicity

Relevant to CNS pathologies, PAFR-mediated increases in intracellular calcium levels and glutamate release can result in excitotoxicity and apoptosis (82, 90, 91). On a tissue level, PAFR are expressed in different regions of the brain including hypothalamus, cerebral cortex, olfactory bulb, hippocampus, brainstem and spinal cord (72). At the cellular level, PAFR is expressed predominantly in microglia, the brain resident immune cells (92, 93), but also found in neurons and other glial cells such as astrocytes and oligodendrocyte progenitor cells (72, 94–96). Though high (micromolar) levels of PAF can certainly induce cell toxicity, PAF acts primarily through PAFR. Indeed, its reported effects can be suppressed in the presence of PAFR antagonists or no longer detected in a PAFR knock-out mouse model. PAF-mediated increase in intracellular calcium concentration (72) and neurotransmitter release (73, 74, 76) are no longer detected in the presence of PAFR antagonists. *In vivo* administration of a PAFR antagonist (LAU-0901) in a mouse model of temporal lobe epilepsy attenuated seizure susceptibility and neuronal hyper-excitability as well as reduced hippocampal damage (82, 96). The adverse CNS effects following TBI were alleviated in mice deficient in PAFR expression (97). Ischemic damage can be attenuated by treatment with PAFR antagonist in rabbits (98). Preclinical studies also showed that PAF signaling through PAFR is involved in the dopaminergic neurodegeneration induced by 1-methyl-4-phenyl-1,2,3,6-tetrahydropyridine (MPTP) as PAFR knockout mice and mice treated with a PAFR antagonist did not suffer neurological deficits from MPTP (99). Furthermore, amyloid beta-induced neurotoxicity can be suppressed by antagonizing PAFR (100, 101).

Although the precise mechanistic steps by which PAF and PAFR play a role in these CNS dysfunction are not yet clearly defined, strong evidence indicates that PAF can induce or enhance neuroinflammation by promoting microglia activation thereby exacerbating disease states and neuronal injury in a PAFR-dependent manner (92, 93). Exogenous application of PAF was shown to induce a microglial chemotactic response that was dependent on PAFR, as chemotaxis was not observed in the presence of PAFR antagonist (WEB2086) or in PAFR deficient mice (102). PAF-mediated activation of microglia is thought to occur through an increase in intracellular calcium concentration (103, 104). The source of the PAF-mediated calcium increase has been reported to be primarily from endoplasmic reticulum stores which in turn induce a more sustained increase in calcium by activation of the store-operated calcium channels (SOCs) (103, 104).

Excessive PAF can also disrupt the blood brain barrier (BBB) *via* a PAFR-dependent mechanism (105–107). Rat brain microvascular endothelial cells, an essential component of the BBB, were shown to have increased PAF production when exposed to hypoxia, consequently resulting in the breakdown of BBB (105). There is evidence that similar to PAF effects on microglia, this PAF-mediated BBB disruption occurs *via* an increase in intracellular calcium (105, 107). However, unlike in microglia, the source of PAF-mediated calcium increase in microvascular endothelial cells is the influx of calcium ions through L-type voltage-gated calcium channels, thereby inducing depolarization, and ultimately resulting in increased BBB permeability (107). Treatment with a PAFR antagonist prevented the PAF-induced increase in calcium concentration and prevented disruption in BBB permeability (105, 107).

### MVP and CNS Pathologies

At present, there are no reports as to the identification of PAF-containing MVP in the CNS. However, microglia release MVP containing IL-1β within the CNS upon ATP stimulation (108). Similar to the formation and release of MVP in other systems, this process in microglia was found to be dependent on aSMase (28). In the case of TBI, mice that were genetically deficient in aSMase or pharmacologically treated with aSMase inhibitor experienced significantly less adverse neurological effects at 1 month post injury (109). It is unclear whether these microparticles also contain PAF or PAFR agonists in addition to IL-1β.

### Potential Pharmacologic Strategies

In summary, multiple studies have implicated the PAF system in a number of neuroinflammatory and neurodegenerative conditions. As these mechanisms are further elucidated, there is potential for PAF and PAFR targeted therapeutics for various CNS disorders. In particular, one area which has significant promise as a therapeutic target relates to MVPs, which are likely elevated in a broad range of CNS pathologies. It is presumed that an enhanced understanding of the PAF system in CNS disorders would be reflected upon the peripheral nervous system.

## PAF and UVB Radiation

### Evidence Linking PAF System to UVB

There is accumulating data suggestive that the PAF system could play a significant role in cutaneous pathophysiology, in particular how the skin responds to exogenous environmental stressors (110). Cell types in the skin such as the keratinocyte (34), mast cell (111, 112), and multiple monocytic and granulocytic cell types express PAFRs (113, 114). Of interest, melanocytes, skin fibroblasts, and T cells do not, yet B cells express PAFRs (34, 115, 116). Elevated PAFR agonist levels can be detected following multiple pathophysiologic stressors ranging from burn injury to X-radiation (41, 117, 118). Moreover, PAF can be detected in cold urticaria (119), immunobullous diseases such as bullous pemphigoid (120), following sunburn (121), and in the skin disease psoriasis (122). Consistent with the notion that PAF could exert cutaneous effects, intradermal injection or topical application of PAF onto skin results in an almost immediate painful urticarial response (68–71).

Ultraviolet B radiation (290-320 nm; UVB) is a pro-oxidative stressor which exerts profound cutaneous effects. Though only appreciably absorbed in the epidermis, sunlight’s “burning rays” are critical for vitamin D metabolism, yet, also generate sunburns as well as both melanoma and non-melanoma skin cancer (8, 9, 123–126). Inasmuch as UVB generates anti-inflammatory effects in skin, phototherapy is used clinically to treat a large number of skin diseases including atopic dermatitis and psoriasis (9)..

Multiple lines of evidence link UVB with the PAF system. UVB generates the production of PAF and ox-GPC with PAFR agonistic activity (14). PAFR signaling is involved in two distinct aspects of photobiology. First, PAFR activation has been implicated in the early acute responses of UVB. In a murine model of photosensitivity from deficiency of the DNA repair enzyme xeroderma pigmentosum type A (XPA), UVB treatment results in dramatically increased levels of ROS, as well as PAF and ox-GPC formation (127). Moreover, UVB-mediated exaggerated pro-inflammatory responses in XPA-deficient mice are blocked by PAFR antagonists (127). Of interest, PAFR-deficient epithelial cells and mice exhibit diminished acute inflammation as well as decreased production of multiple cytokines including tumor necrosis factor in response to UVB (71, 127–129). Second, UVB-mediated systemic immunosuppression involves PAFR signaling *via* ox-GPCs (6–9, 112). Of note, UVB induces both local immunosuppression (where a UVB-treated site is anergic) in addition to systemic immunosuppression (where a non-UVB-treated UVB site is anergic). UVB-mediated local immunosuppression appears to involve dendritic cells, whereas systemic immunosuppression involves mast cells and Tregs. It has been reported by several groups that exogenous PAF is immunosuppressive, and UVB-induced systemic immunosuppression is attenuated by PAFR antagonists and absent in PAFR deficient mice (6–9, 112). However, local immunosuppression is normal in PAFR KO mice (129). As noted elsewhere in this review, exogenous PAF agonists augment experimental melanoma tumor progression (41–43). Finally, systemic PAFR antagonists have been reported to inhibit tumor formation in a murine model of UVB photocarcinogenesis (130).

### PAF and UVB and MVP

Environmental stressors which produce PAF such as thermal burn injury or UVB also cause the production and release of MVP (24, 52, 131, 132). Indeed, MVP generated in response to UVB or thermal burn injury by keratinocytes *in vitro*, human skin *ex vivo*, and murine skin *in vivo* is dependent upon the PAFR (24, 25). Finally, MVP produced in response to UVB or thermal burn injury contain PAFR agonistic activity (24).

**Figure 1:**
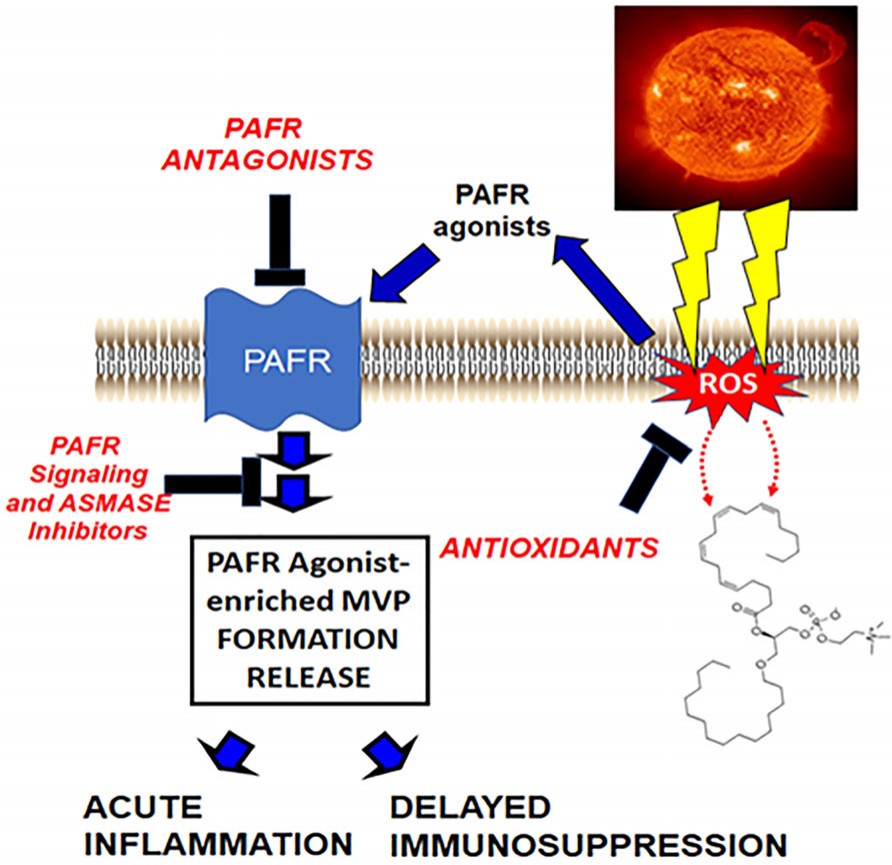
Hypothetical model by which UVB generates PAFR agonists *via* ROS which then result in PAF-laden MVP release. In this model, ROS generated by UVB result in ox-GPC as well as enzymatic PAF synthesis. These PAFR agonists act upon the PAFR resulting in MVP generation release *via* acid sphingomyelinase activation. These MVP contain bioactive agents, especially PAFR agonists which then can mediate UVB effects.

The picture that is emerging (see **Figure 1**) fits with the hypothesis that UVB generates ox-GPC PAFR agonists, which in turn act upon the PAFR-positive keratinocyte resulting in PAFR activation. This PAFR activation then results in additional PAF enzymatically, as well as increased ROS and thus more ox-GPC. PAFR activation also translocates aSMase which then triggers MVP release. The MVP which contain PAF and ox-GPC then travel from the epidermis. One of the critical PAFR cell types these UVB-generated MVP (UVB-MVP) encounter is the dermal mast cell, which contributes to both UVB acute responses as well as delayed systemic immunosuppression.

### Potential Pharmacologic Strategies

Knowledge gaps which need to be addressed include the role the PAF system and UVB-MVP in photosensitivity, as well as in photocarcinogenesis. Of note, a human study using the COX-2 inhibitor celecoxib demonstrated decreased numbers of skin cancers (133), which could be in part related to the ability of these agents to block UVB-mediated systemic immunosuppression (6–9). Since MVP generation and release can be modulated pharmacologically by aSMase inhibitors such as imipramine (25, 29), addressing these knowledge gaps could result in novel strategies for managing photodermatology disorders and photocarcinogenesis.

## Discussion

Though much has been learned about this class of lipid mediators first described almost 50 years ago, significant knowledge gaps remain. Several major hurdles have limited our understanding of the PAF system. First, there is a tremendous heterogeneity of GPC species produced enzymatically and even more *via* non-enzymatic processes which can exert PAFR agonistic effects (5, 15). Moreover, some members of the PAF family of mediators include the 1-acyl species which are in much greater abundance than 1-alkyl GPC, yet have much lower binding affinities and have been suggested to act as antagonists (120, 134, 135). PAF is rapidly metabolized, with a half-life in biological fluids measured in minutes. Hence, it is challenging to be able to accurately measure PAFR activity in biologic systems. A second issue that potentially limits the study of, and ability to use pharmacologic tools is also that PAFR antagonists are of much lower affinity than the most active PAF species, and that inhibitors of the biosynthetic and inactivation enzymatic pathways are not available.

A relatively new area which could result in an enhanced understanding of the PAF system relates to how PAF is released from a host cell. Extracellular vesicles such as MVP appear to be a logical mechanism for the release of PAF and multiple bioactive molecules. Recent findings from our group indicate that PAFR activation is a potent mediator for MVP generation/release, and that these MVP contain PAFR agonistic activity (24). Inasmuch as MVP formation can be blocked by aSMase inhibitors, this allows another potential level of pharmacologic intervention. Future studies should provide new insights into the PAF system which should result in novel targets for diseases of the skin as well as for other systemic disorders.

## Author Contributions 

JT wrote large parts of the manuscript. JR and RS wrote parts of the manuscript and proofread. All authors contributed to the article and approved the submitted version.

## Conflict of Interest

JR is an employee of the U.S. Government. This work was prepared as part of her official duties. Title 17, USC, §105 provides that ‘Copyright protection under this title is not available for any work of the U.S. Government.’ Title 17, USC, §101 defines a U.S. Government work as a work prepared by a military service member or employee of the U.S. Government as part of that person’s official duties.

This research was supported in part by grants from the National Institutes of Health grant R01 HL062996 (JT), Veteran’s Administration Merit Award 5I01BX000853 (JT).

The remaining authors declare that the research was conducted in the absence of any commercial or financial relationships that could be construed as a potential conflict of interest. The views expressed in this presentation are those of the authors and do not necessarily reflect the official policy or position of the Department of the Navy, Department of Defense, nor the U.S. Government.
